# Diffusion-weighted imaging-based radiomics for predicting 1-year ischemic stroke recurrence

**DOI:** 10.3389/fneur.2022.1012896

**Published:** 2022-10-28

**Authors:** Hao Wang, Yi Sun, Jie Zhu, Yuzhong Zhuang, Bin Song

**Affiliations:** Department of Radiology, Minhang Hospital, Fudan University, Shanghai, China

**Keywords:** stroke, recurrence, radiology, magnetic resonance imaging, diffusion

## Abstract

**Purpose:**

To investigate radiomics based on DWI (diffusion-weighted imaging) for predicting 1-year ischemic stroke recurrence.

**Methods:**

A total of 1,580 ischemic stroke patients were enrolled in this retrospective study conducted from January 2018 to April 2021. Demographic and clinical characteristics were compared between recurrence and non-recurrence groups. On DWI, lesions were segmented using a 2D U-Net automatic segmentation network. Further, radiomics feature extraction was done using the segmented mask matrix on DWI and the corresponding ADC map. Additionally, radiomics features were extracted. The study participants were divided into a training cohort (*n* = 157, 57 recurrence patients, and 100 non-recurrence patients) and a test cohort (*n* = 846, 28 recurrence patients, 818 non-recurrence patients). A sparse representation feature selection model was performed to select features. Further classification was accomplished using a recurrent neural network (RNN). The area under the receiver operating characteristic curve values was obtained for model performance.

**Results:**

A total of 1,003 ischemic stroke patients (682 men and 321 women; mean age: 65.90 ± 12.44 years) were included in the final analysis. About 85 patients (8.5%) recurred in 1 year, and patients in the recurrence group were older than the non-recurrence group (*P* = 0.003). The stroke subtype was significantly different between recurrence and non-recurrence groups, and cardioembolic stroke (11.3%) and large artery atherosclerosis patients (10.3%) showed a higher recurrence percentage (*P* = 0.005). Secondary prevention after discharge (statins, antiplatelets, and anticoagulants) was found significantly different between the two groups (*P* = 0.004). The area under the curve (AUC) of clinical-based model and radiomics-based model were 0.675 (95% CI: 0.643–0.707) and 0.779 (95% CI: 0.750–0.807), respectively. With an AUC of 0.847 (95% CI: 0.821–0.870), the model that combined clinical and radiomic characteristics performed better.

**Conclusion:**

DWI-based radiomics could help to predict 1-year ischemic stroke recurrence.

## Introduction

Stroke remains a leading cause of high disability rate and mortality in the world, accounting for 9–20% of around 10 million strokes that occur each year (1, 2). About 80% of all stroke cases are caused by ischemic stroke (3). According to previous studies, recurrent stroke rates in the western country ranged from 10 to 17% (4). Higher stroke recurrence was reported in Asian populations, ranging from 11.2 to 18.9% in China and Japan (4, 5). One-year recurrence rate was reported in a systematic review and meta-analysis ranged from 5.7 to 17.7% (6).

Studies suggested that a higher risk of disability, dementia, and mortality are associated with recurrent stroke comparing first-time stroke, which might indicate the failure of medical therapy (7–9). Trends and risks of stroke recurrence (10), secondary stroke prevention strategies (4, 11), and demographic characteristics (2) that were evaluated in stroke patients have all been examined in previous studies. Some studies reported laboratory tests, such as peripheral immune cells including neutrophil and lymphocyte counts, infection (12), genetic variants (13), and low-density lipoprotein cholesterol levels (14), were associated with stroke recurrence. In addition, to predict recurrence, artery stenosis, atherosclerotic plaque features, and artery wall change were studied (5, 15–18). Magnetic resonance imaging (MRI) plays an important role in the diagnosis and prognosis of ischemic stroke. However, few studies focused on ischemic stroke lesions based on imaging in predicting stroke recurrence (19, 20). Only a few low-order features of diffusion-weighted imaging (DWI), such as lesion number and presence of lesions, were assessed.

Radiomics is an emerging non-invasive method of extracting high-throughput quantitative features for predicting important clinical outcomes. A previous study demonstrated that radiomics features based on DWI showed good performance in predicting clinical functional outcomes in ischemic stroke patients (3). A recent study that enrolled 522 acute ischemic stroke (AIS) patients showed that the clinical-radiomics model outperformed individual clinical or radiomics models in predicting AIS outcomes (21). To the best of our knowledge, no studies explored the stroke recurrence risk using radiomics features based on DWI. Therefore, we sought to explore DWI-based radiomics for predicting stroke recurrence in 1 year.

## Methods

### Study population

From January 2018 to April 2021, patients with first-ever ischemic stroke were enrolled in this retrospective study. This study was conducted at our stroke center. The retrospective study had approval from the Institutional Ethics Committee of our hospital (approval number: 2022-013-01 K). Moreover, patients provided written informed consent before MRI examinations. The study was performed in accordance with the 1964 Declaration of Helsinki and its later amendments.

*The exclusion criteria were as follows:*

Patients with cerebral hemorrhage, traumatic brain injury, previous neurological or psychiatric disorder, cerebral tumor, a history of substance abuse, severe MRI artifacts, a contradiction to MR examination, inability to extract effective features due to the too mall lesions size (<50 voxels), or lost to follow-up were excluded from the study.

**Figure 1:**
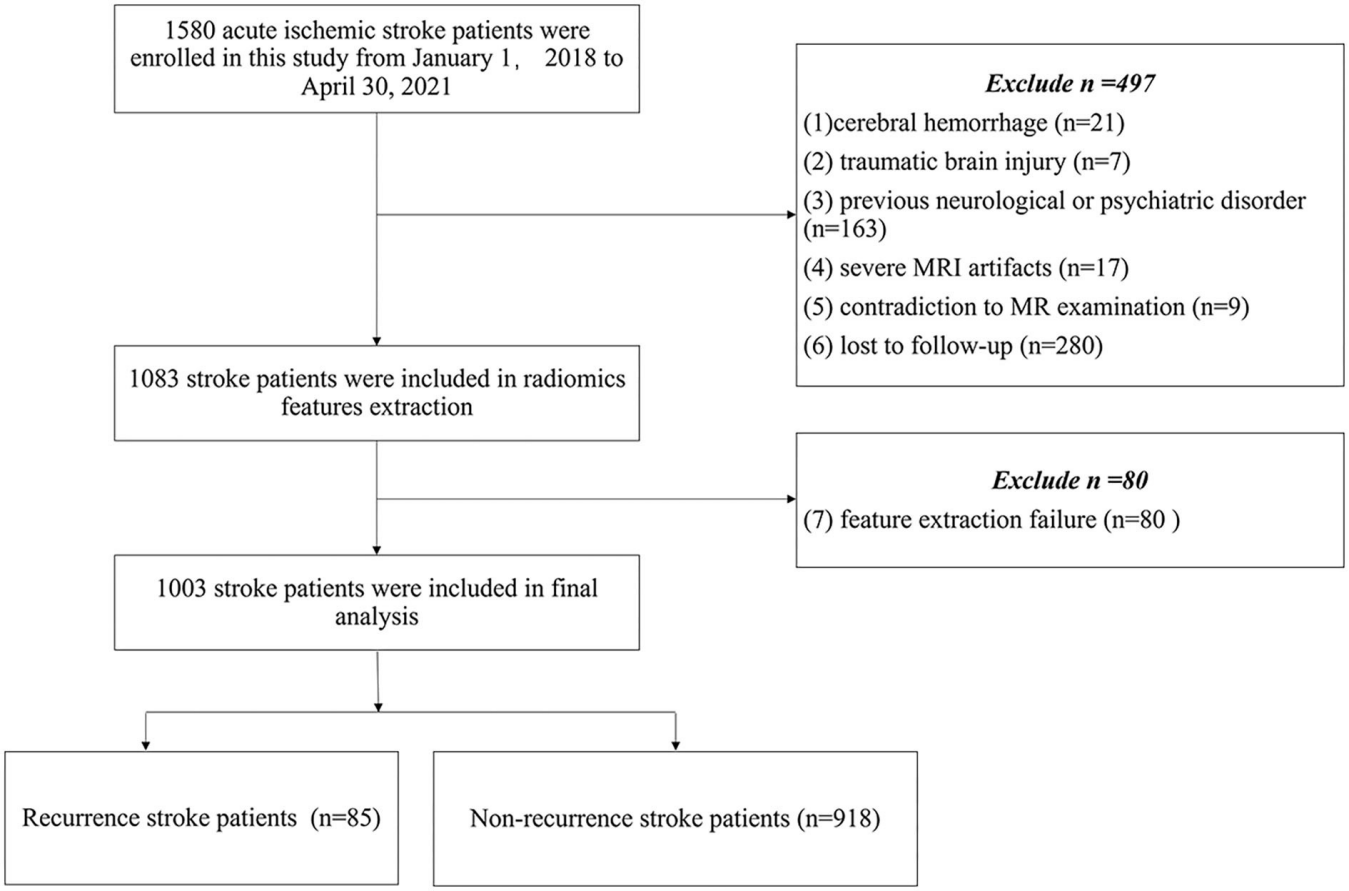
Flow chart outlines study group selection.

The demographic and clinical characteristics were recorded, including age, sex, smoking, drinking, hypertension, hyperlipidemia, diabetes mellitus, atrial fibrillation, the National Institutes of Health Stroke Scale (NIHSS) score on admission, Trial of Org 10172 in Acute Stroke Treatment (TOAST) stroke subtype classification (22), secondary prevention after discharge (antiplatelets, anticoagulants, or statins), a territory of circulation (anterior, posterior, both anterior and posterior), and modified Rankin Scale (mRS) at 90 days. The study flow chart is shown in Figure 1.

### Outcome assessments

A recurrent ischemic stroke was defined as a new sudden focal neurological deficit occurring at any time after discharge and confirmed on DWI. In all cases with event recurrence, the date of ischemic event recurrence was ascertained by face-to-face interviews or telephone interviews and confirmed by a review of hospital records. A consensus choice regarding stroke progression or recurrence was made after a thorough examination of these outcome events by one stroke neurologist and one neuroradiologist.

### MR acquisition

On scanner 1, an MRI was obtained (EXCITE HD 1.5 T MRI; GE Healthcare, Milwaukee, WI, USA) comprising a 16-channel head/neck coil; and scanner 2 (uMR780 3.0 T MRI; United Imaging Healthcare, Shanghai, China) was equipped with a 24-channel head/neck coil. The detailed scan parameter includes axial fluid-attenuated inversion recovery, DWI including apparent diffusion coefficient (ADC) maps, and T2-weighted and T1-weighted sequences (Supplementary Table e-1). All patients underwent MRI scans within 72 h of symptom onset.

### Segmentation of infarction lesions and image preprocessing

**Figure 2:**
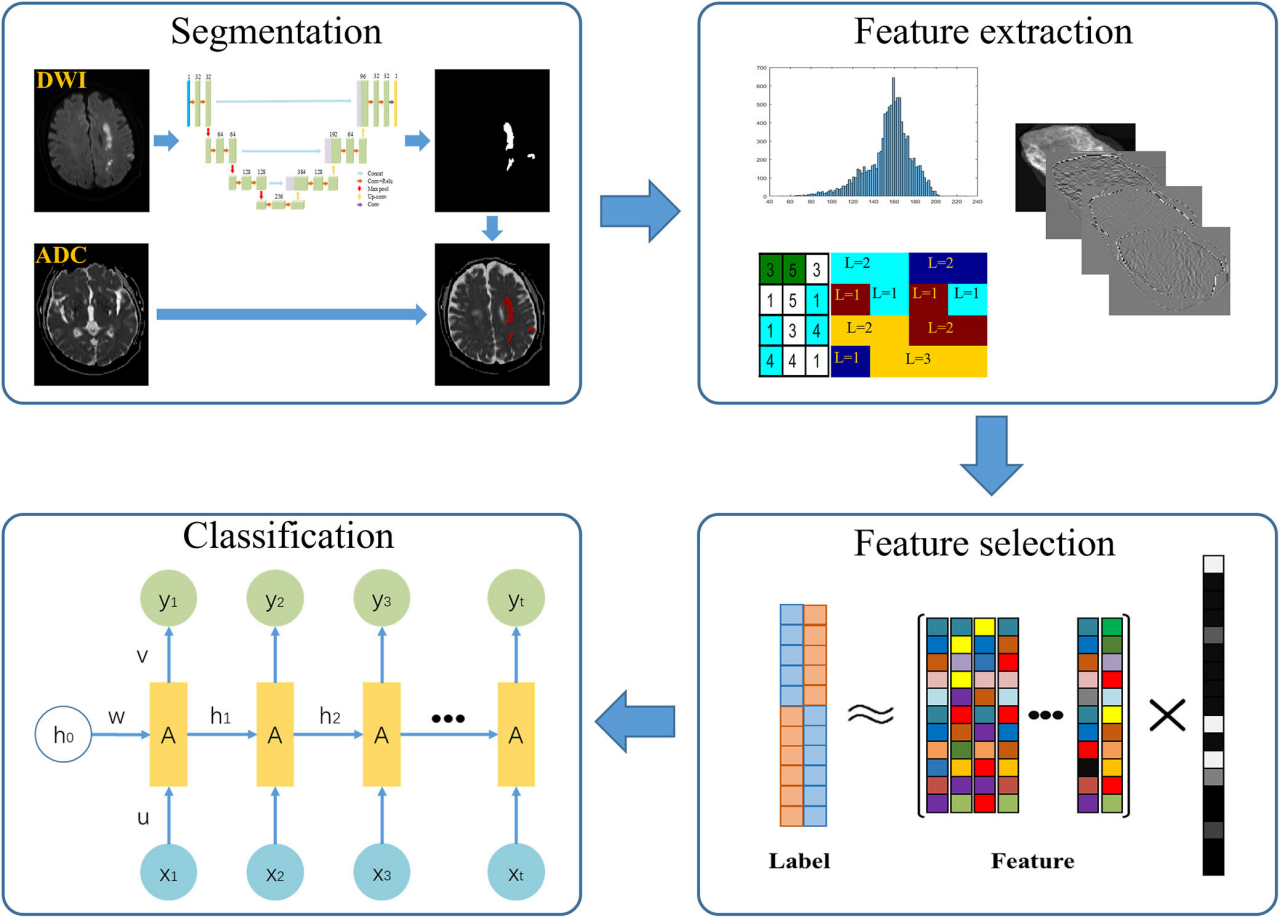
Pipeline of radiomics analysis of ischemic stroke on diffusion-weighted imaging.

First, we randomly selected 100 patients. With ITK-SNAP (http://www.itk-snap.org), infarction lesions were manually segmented by two senior radiologists. Slice-by-slice stacking of DWI images by one neuroradiologist defined the 3D volume of interest of each infarct lesion. Then, we trained 2D U-Net automatic segmentation network on the manually segmented 100 volumes (23, 24). We further segmented all cases based on the trained segmentation network model. Radiomics features were extracted from the ADC map using the segmented mask matrix on corresponding DWI (Figure 2). GitHub (https://github.com/wuguoqingfudan/stroke-segmentation.git) received the automatic segmentation code. In addition, we randomly selected 100 participants segmented by an experienced neuroradiologist and tested the performance of automatic segmentation using the DICE coefficient.

We randomly selected 100 cases from the testing set and calculated the DICE coefficient between the radiologist's annotation results and the network's automatic segmentation results. Before the process of training and testing the network-based image segmentation, image intensity parameters were normalized to 0–1 using window width and window level. Before radiomics feature extraction, we deleted the partial segmentation area (pixel < 20), which is hard to reflect texture features of infarct lesions.

### Radiomics feature extraction and selection

First, intensity- and texture-related features were extracted from the ADC map in the image domain. Second, these features were extracted using wavelet domain images generated by performing an 8-wavelet transform on the origin images. Feature extraction was performed on the MATLAB software (MathWorks) (25) and referenced literature (26). The Supplementary Table e-2 provided more information about the extracted features.

A sparse representation feature selection model was used to select a small number of high discriminative features to reduce the redundancy of extracted features.

The equation feature selection model was as follows:


$$\hat{w}-\arg_{w}\min||l-Fw|{|}_{2}^{2}+\eta ||w|{|}_{0}$$

(27).

Where, *l*∈*R*^*m*^ represents the label of the training sample,*m* represents the number of the training sample, $F={\left[f_{1},f_{2},...f_{m}\right]}^{T}\in R^{m\times 2K}$ represents the set of features of the training sample, η represents the control parameter of sparse representation, and the absolute values of the coefficient *w* corresponding to the importance of features. When *w* is obtained, the key features can be selected by simple threshold comparison.

### Data distribution and classification

**Figure 3:**
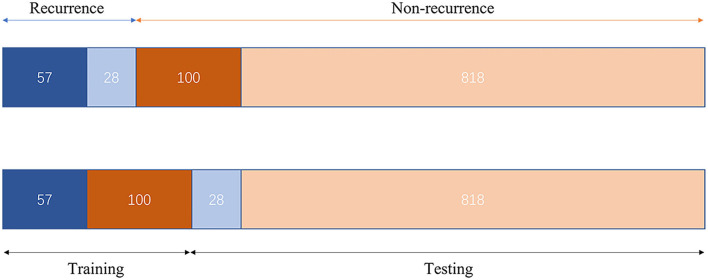
Training and testing cohort data distribution.

The study participants were divided into two cohorts, that is, the training cohort and the testing cohort. Further classification was accomplished using a recurrent neural network (RNN) (28). Due to the significant difference in the proportion of positive and negative samples in our study [85 recurrences vs 918 non-recurrences, the proportion was consistent with some previous studies (10, 20)], we used a weighted cross-entropy loss function to optimize the network. Furthermore, we constructed the training cohort with an under-sampling approach in terms of model training data. The training cohort included 57 recurrence patients and 100 non-recurrence patients. The remaining 846 patients were included in the test cohort to confirm the classification's accuracy. The Adam optimizer was used for in-network training, with the learning rate set to 0.0001 and the batch size set to 10. Figure 3 depicts the data distribution. PyTorch was used to create the suggested model. The entire training was carried out on a computer with an Intel Xeon Gold 6128 CPU with 64 GB RAM and an Nvidia TITAN Xp (12 GB).

### Statistical analysis

All the statistical analyses were performed with SPSS (version 23.0, SPSS Inc., Chicago, IL, USA). Continuous variables with normal distribution were represented by mean ± standard deviation, non-normally distributed variables by median (interquartile range), and classification variables by frequency (%). We used the Student *t*-test or the Mann–Whitney *U* test for continuous and the χ^2^ test for categorical dependent variables (or Fisher's exact test where appropriate) between recurrence and non-recurrence groups.

The receiver-operative curve (ROC) was performed to compare the performance of clinical characteristics and the selected radiomics features in the evaluation of stroke recurrence. The area under the ROC curve (AUC) was constructed by plotting the true-positive rate against the false-positive rate for different binary classification thresholds of the predictors. All P-values were calculated using two-tailed tests, and *P* < 0.05 was considered statistically significant. In addition, we compared the performance of each model using the Delong test. The decision curve analysis (DCV) was implemented to assess the clinical value of the predictive models.

## Results

### Demographic and clinical characteristics

The final study enrolled 1,003 patients (682 men and 321 women; mean age: 65.90 ± 12.44 years) with acute ischemic stroke. The following factors led to the exclusion of 577 patients: cerebral hemorrhage (*n* = 21); (2) traumatic brain injury (*n* = 7); (3) previous neurological or psychiatric disorder (*n* = 163); (4) severe MRI artifacts (*n* = 17); (5) contradiction to MR examination (*n* = 9); (6) lost to follow-up (*n* = 280); and (7) inability to extract effective features (*n* = 80). The final analysis included 1003 patients (mean age: 65.90 ± 12.44). The clinical and demographic characteristics are summarized in Table 1. Of the 1,003 patients, 85 patients (8.5%) recurred in 1 year, and recurrence stroke patients were older than non-recurrence stroke patients (*P* = 0.003). The stroke subtype was a significant difference between recurrence and non-recurrence groups, and cardioembolic stroke showed a higher recurrence percentage in our study (*P* = 0.005). Secondary prevention after discharge was found significantly different between the two groups. Statins and anticoagulants showed higher recurrence in secondary prevention after discharge in our study (*P* = 0.004 and *P* = 0.02, respectively). There were no significant differences in gender, smoking, drinking, hypertension, hyperlipidemia, diabetes mellitus, atrial fibrillation, circulation territory, and NIHSS at admission and mRS at 90 days between non-recurrence and recurrence stroke patients (all *P* > 0.05).

**Table 1:** Demographic and clinical characteristics in acute ischemic stroke patients.

**Variables**		**Non-recurrence** **(*n* = 918, 91.5%)**	**Recurrence** **(*n* = 85, 8.5%)**	***P***
Age	/	65.55 ± 12.35	69.74 ± 12.79	0.003
Gender	Male	626 (91.8%)	56 (8.2%)	0.662
	Female	292 (91.0%)	29 (9.0%)	
Smoking		578 (92.5%)	47 (7.5%)	0.163
	Yes	340 (89.9%)	38 (10.1%)	
Drinking		791 (91.0%)	78 9.0%)	0.147
	Yes	127 (94.8%)	7 (5.2%)	
Hypertension		296 (90.5%)	31 (9.5%)	0.426
	Yes	622 (92.0%)	54 (8.0%)	
Hyperlipidemia		662 (91.2%)	64 (8.8%)	0.530
	Yes	256 (92.4%)	21 (7.6%)	
Diabetes mellitus		596 (91.3%)	57 (8.7%)	0.693
	Yes	322 (92.0%)	28 (8.0%)	
Atrial fibrillation		817 (91.8%)	73 (8.2%)	0.385
	Yes	101 (89.4%)	12 (10.6%)	
Stroke subtype (TOAST)	Large artery atherosclerosis	488 (89.7%)	56 (10.3%)	0.024
	Cardioembolic stroke	71 (88.8%)	9 (11.3%)	
	Small artery occlusion	280 (94.0%)	18 (6.0%)	
	Other cause	13 (100.0%)	0 (0.0%)	
	Undetermined cause	66 (97.1%)	2 (2.9%)	
Circulation territory	Anterior	629 (91.4%)	59 (8.6%)	0.602
	Posterior	263 (92.3%)	22 (7.7%)	
	Both	26 (86.7%)	4 (13.3%)	
Statins after discharge		350 (94.9%)	19 (5.1%)	0.004
	Yes	568 (89.6%)	66 (10.4%)	
Antiplatelets after discharge		92 (86.8%)	14 (13.2%)	0.064
	Yes	826 (92.1%)	71 (7.9%)	
Anticoagulants after discharge		881 (92.0%)	77 (8.0%)	0.047
	Yes	37 (82.2%)	8 (17.8%)	
NIHSS at admission	/	3 (1,4)	3 (1,4)	0.792
mRS at 90 days	/	1 (0,2)	1 (0,1)	0.908
Symptom onset to DWI scan time (hours)	/	33.56 ± 65.09	39.89 ± 60.25	0.721

The bold values indicate the value of *p* less than 0.05.

### Difference of stroke subtype in stroke recurrence

Table 2 provides a summary of the demographic and clinical traits of the three primary non-recurrence and recurrence stroke subtypes. The mean interval time between the first stroke and stroke recurrence was 167.11 ± 100.08 days.

**Table 2:** Demographic and clinical characteristics of different ischemic stroke subtypes (TOAST).

**Variables**		**Large artery atherosclerosis**			**Small artery occlusion**			**Cardioembolic stroke**		
		**Non-recurrence (*n* = 488, 89.7%)**	**Recurrence (*n* = 56, 10.3%)**	***P***	**Non-recurrence (*n* = 280, 94.0%)**	**Recurrence (*n* = 18, 6.0%)**	***P***	**Non-recurrence (*n* = 71, 88.7%)**	**Recurrence (*n* = 9, 11.3%)**	***P***
Age	/	65.57 ± 12.09	69.73 ± 13.16	0.016	63.80 ± 12.25	73.39 ± 8.60	< 0.001	73.21 ± 11.048	64.44 ± 15.88	0.036
Gender	Male	330 (90.9%)	33 (9.1%)	0.191	202 (93.5%)	14 (6.5%)	0.787	42 (85.7%)	7 (14.3%)	0.470
	Female	158 (87.3%)	23 (12.7%)		78 (95.1)	4 (4.9%)		29 (93.5)	2 (6.5%)	
Smoking	No	319 (90.9%)	32 (9.1%)	0.223	234 (93.6%)	16 (95.8%)	0.747	51 (94.4%)	3 (5.6%)	0.052
	Yes	169 (87.6%)	24 (12.4%)		46 (6.4%)	2 (4.2%)		20 (76.9%)	6 (23.1)	
Drinking	No	419 (89.1%)	51 (10.9%)	0.281	162 (93.1%)	12 (95.2%)	0.462	66 (88.0%)	9 (12.0)	>0.999
	Yes	69 (93.2%)	5 (6.8%)		118 (6.9%)	6 (4.8%)		5 (100.0%)	0 (0.0%)	
Hypertension	No	158 (88.8%)	20 (11.2%)	0.614	92 (93.9%)	6 (6.1%)	0.967	18 (78.3%)	5 (23.7%)	0.111
	Yes	330 (90.2%)	36 (9.8%)		188 (94.0%)	12 (6.0%)		53 (93.0%)	4 (7.0%)	
Hyperlipidemia	No	342 (88.8%)	43 (11.2%)	0.296	212 94.2%)	13 (5.8%)	0.778	56 (88.9%)	7 (11.1%)	>0.999
	Yes	146 (91.8%)	13 (8.2%)		68 (93.2%)	5 (6.8%)		15 (88.2%)	2 (11.8%)	
Diabetes mellitus	No	328 (88.9%)	41 (11.1%)	0.363	174 (94.1%)	11 (5.9%)	0.930	54 (93.1%)	4 (6.9%)	0.105
	Yes	160 (91.4%)	15 (8.6%)		106 (93.8%)	7 (6.2%)		17 (77.3%)	5 (22.7%)	
Atrial fibrillation	No	453 (91.1%)	44 (8.9%)	0.001	274 (93.8%)	18 (6.2%)	>0.999	21 (70.0%)	9 (30.0%)	< 0.001
	Yes	35 (74.5%)	12 (25.5%)		6 (100.0%)	0 (0.0%)		50 (100.0%)	0 (0.0%)	
Statins after discharge	No	185 (94.4%)	11 (5.6%)	0.007	109 (96.5%)	4 (92.4%)	0.157	22 (84.6%)	4 (15.4%)	0.462
	Yes	303 (87.1%)	45 (12.9%)		171 (3.5%)	14 (7.6%)		49 (90.7%)	5 (9.3%)	
Antiplatelets after discharge	No	38 (80.9%)	9 (19.1%)	0.045	14 (82.4%)	3 (17.6%)	0.074	24 (96.0%)	1 (4.0%)	0.260
	Yes	450 (90.5%)	47 (9.5%)		266 (94.7%)	15 (5.3%)		47 (85.5%)	8 (14.5%)	
Anticoagulants after discharge	No	476 (90.8%)	48 (9.2%)	< 0.001	278 (93.9%)	18 (6.1%)	>0.999	52 (85.2%)	9 (14.8%)	0.160
	Yes	12 (60.0%)	8 (40.0%)		2 (100.0%)	0 (0.0%)		19 (100.0%)	0 (0.0%)	
NIHSS	/	3 (2, 5)	3 (1, 4)	0.298	2 (1, 3)	2 (1, 4)	0.192	4 (2, 7)	2 (2, 5)	0.433
mRS at 90 days	/	1 (0, 2)	1 (0, 2)	0.687	0 (0, 1)	0 (0, 1)	0.921	0 (0, 2)	0 (0, 0)	0.252

TOAST, Trial of Org 10172 in Acute Stroke Treatment; NIHSS, National Institutes of Health Stroke Scale. The bold values indicate the value of *p* less than 0.05.

#### Large artery atherosclerosis

Of the 544 large artery atherosclerosis (LAA) patients (362 men and 181 women; mean age: 65.99 ± 12.26 years), 56 (10.3%) patients were found to have a recurrence in 1 year, and recurrence stroke patients were also older than non-recurrence stroke patients (*P* = 0.016). After discharge, stroke patients who did not use antiplatelets had a higher rate of recurrence than those who did (*P* = 0.045). On the contrary, stroke recurrence was found more in stroke patients using statins and anticoagulants after discharge than those without using statins (*P* = 0.007) and anticoagulants (*P* < 0.001). Atrial fibrillation showed more recurrence of stroke in LAA stroke patients (*P* = 0.001). Gender, smoking, drinking, hypertension, hyperlipidemia, diabetes mellitus, NIHSS at admission, and mRS at 90 days were not found significant differences between the non-recurrence and recurrence stroke patients (all *P* > 0.05).

#### Small artery occlusion

Our research found 298 patients (216 men and 82 women; mean age: 64.38 ± 12.27 years) with small artery occlusion (SAO). Of the 298 SAO patients, 18 (6.0%) patients were found to have a recurrence in 1 year, recurrence stroke patients were also older than non-recurrence stroke patients (*P* < 0.001). Gender, smoking, drinking, hypertension, hyperlipidemia, diabetes mellitus, atrial fibrillation, NIHSS at admission, and mRS at 90 days were not found significant differences between the non-recurrence and recurrence stroke patients (all *P* > 0.05).

#### Cardioembolic stroke

Our study found a total of 80 cardioembolic stroke patients (49 men and 31 women; mean age: 72.22 ± 11.89 years). Nine patients (11.3%) out of the 80 patients had a recurrence within a year. Out of these, recurrence stroke patients were younger than non-recurrence stroke patients (*P* = 0.036). Atrial fibrillation showed more non-recurrence stroke in cardioembolic stroke patients (*P* < 0.001). Gender, smoking, drinking, hypertension, hyperlipidemia, diabetes mellitus, NIHSS at admission, and mRS at 90 days were not found significant differences between the non-recurrence and recurrence stroke patients (all *P* > 0.05).

### The performance of segmentation of infarction

The DICE coefficient between the radiologist's annotation results and the network's automatic segmentation results is 0.886.

### Radiomics feature extraction and selection

A total of 513 radiomics features were extracted, including intensity-related features (18), texture features (39), and wavelet features [8 × (18 + 39)]. Finally, a total of 100 features were selected using a sparse representation feature selection model, that is, clinical characteristics (*n* = 6), image domain features (*n* = 8), and wavelet domain features (*n* = 86) (more feature selection was shown in the Supplementary Table e-3).

### Performance of prediction model

**Figure 4:**
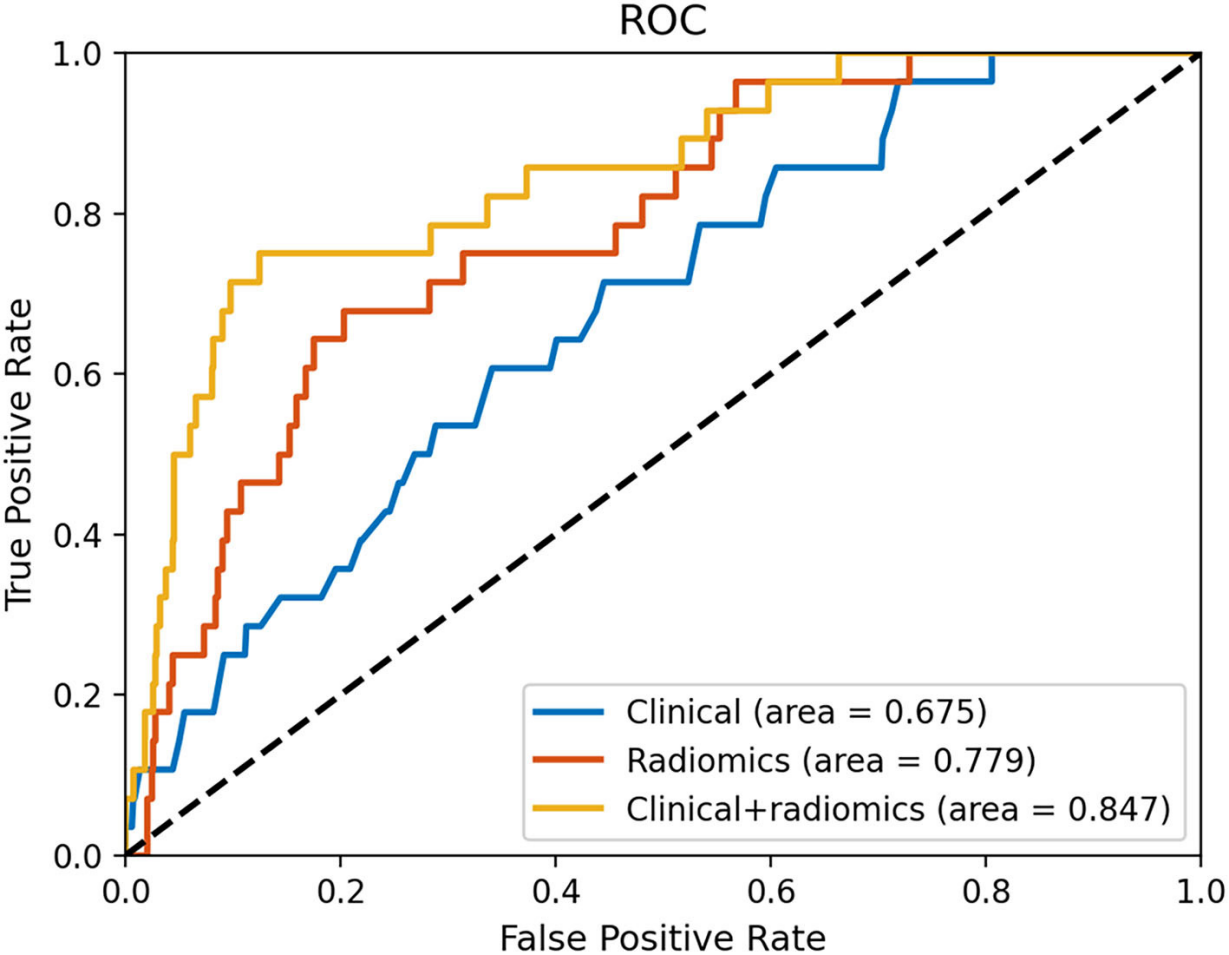
Receiver operating characteristic curves based on the clinical characteristics and radiomics.

**Figure 5:**
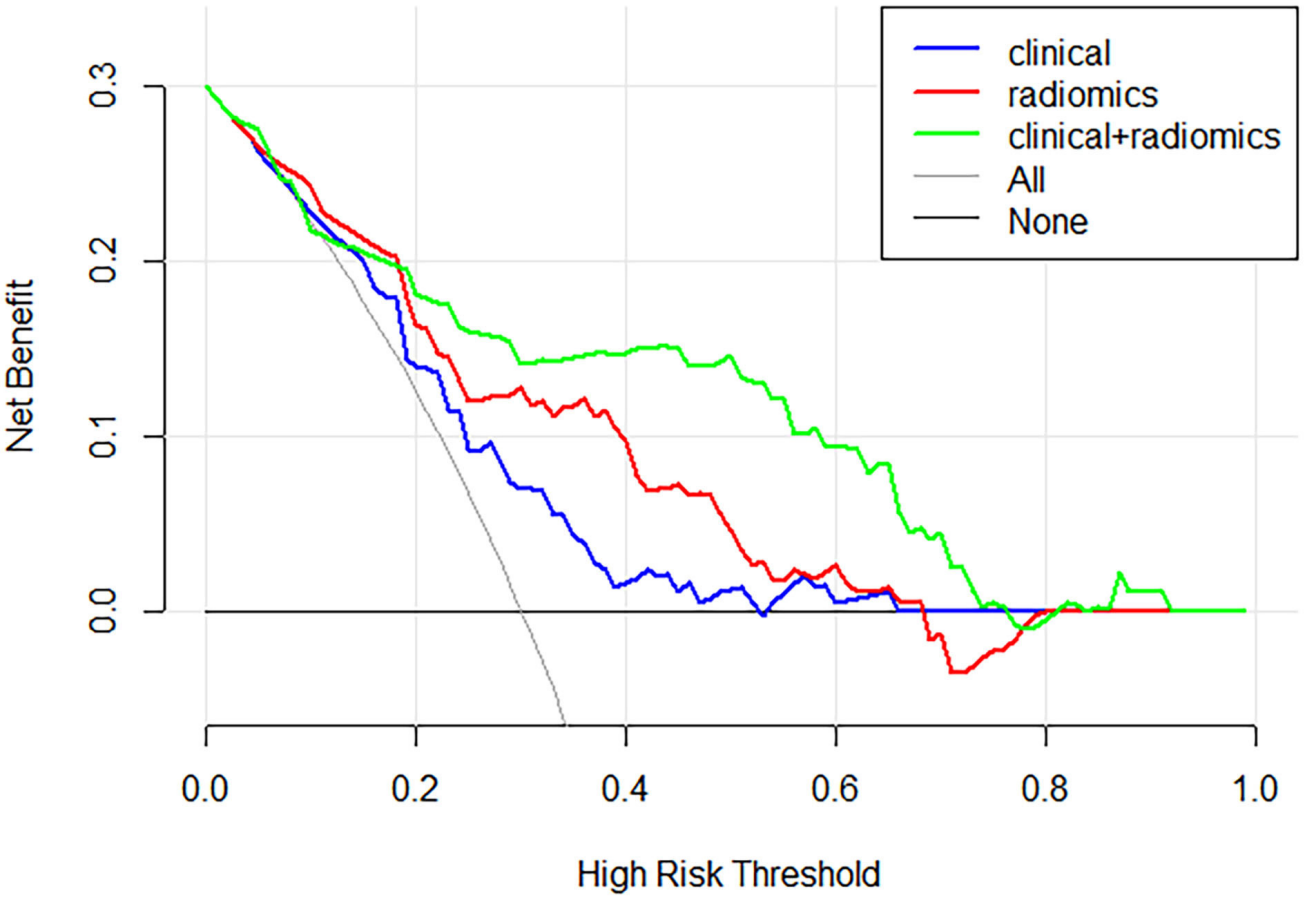
Decision curve analysis for each model. The combination model had the highest net benefit compared with both the other models.

Figure 4 and Table 3 show the performance of the clinical and radiomics-based prediction model. The area under the curve (AUC) of the clinical-based model and radiomics-based models were 0.675 (0.643–0.707) and 0.779 (0.750–0.807), respectively. With an AUC of 0.847 (0.821–0.870), the model that incorporated both clinical and radiomic characteristics outperformed the model that only used clinical characteristics. The combined model presented showed better than clinical-based model (*P* = 0.001) and radiomics-based model (*P* = 0.2), and radiomics-based model was better than clinics-based model (*P* = 0.01). The combination model had the highest net benefit compared with both the other models (Figure 5).

**Table 3:** Performance of two different prediction models.

	**AUC (95% CI)**	**Accuracy**	**Sensitivity**	**Specificity**
Clinical	0.675 (0.643-0.707)	0.670	0.536	0.675
Radiomics	0.779 (0.750-0.807)	0.792	0.679	0.796
Clinical+ radiomics	0.847 (0.821-0.870)	0.833	0.750	0.836

## Discussion

Our study shows that a radiomics prediction model based on stroke infarction lesions derived from the ADC map can accurately predict ischemic stroke recurrence. In this study, we used a 2D U-Net automatic segmentation network based on manually segmented volumes to implement infarction automatic segmentation. In addition, a sparse representation feature selection model was performed to select a small number of high-resolution features to construct the robust prediction model.

A recent study showed that DWI-positive infarction lesion was associated with higher recurrent risk in patients with minor ischemic stroke (MIS) and transient ischemic attack (TIA) (20). Previous studies indicated that large artery atherosclerosis and infarction number were independent predictors of 1-year stroke recurrence with TIA or MIS. Multiple acute infarctions are usually related to embolic pathogenesis (29, 30), which could indicate a relatively unstable cause associated with higher recurrence (31, 32). The majority of patients with a single acute lacunar infarction have pathological changes like fibrinoid necrosis, lipohyalinosis, or other unknown changes. We hypothesized that different stroke subtypes with different pathologic mechanisms could contribute to different recurrent risks. Radiomics can convert medical images into high-throughput quantitative features, mainly comprising histogram and texture features, which have been widely applied in predicting clinical prognosis, pathological grading, and response to treatment (3). The ischemic cascade, which included the production of reactive oxygen species, the release of glutamate, the buildup of intracellular calcium, and the induction of inflammatory processes, is the name given to the pathophysiological mechanisms underlying ischemic stroke. A previous study showed that the texture features based on DWI were closely related to edema after cerebral infarction (33).

In terms of clinical and demographic characteristics, it is not surprising that recurrence stroke patients were older than non-recurrence patients. Different stroke subtypes presented significantly different stroke recurrence incidences. LAA and cardioembolic stroke showed higher recurrence compared to the other subtypes in this study, which was in accordance with the previous study (34). We further analyzed the risk factors of different stroke subtypes in predicting stroke recurrence. After discharge secondary prevention such as statins, anticoagulants, and antiplatelets showed significant differences between recurrent and non-recurrent stroke patients in our study. Antiplatelets could effectively reduce the risk of stroke recurrence in LAA stroke patients. A previous study also demonstrated that less antiplatelet at discharge was associated with a higher recurrence rate (20). Another study showed that there was no evidence of heterogeneity in the effects of antiplatelet therapy by the presence of DWI-positive infarctions vs. absence on the risk of any recurrent stroke (19). Patients with non-recurrence and recurrent strokes did not have significantly different antiplatelet levels, which may be related to the disease's heterogeneity (14). However, the multivariate regression analysis clinical prediction model had a poor performance in our study, with an AUC of 0.675.

In a previous study, imaging parameters (LAA and positive neuroimaging findings) were associated with a higher risk of recurrent stroke rather than clinical characteristics (20). In their study, DWI-positive lesion was associated with higher recurrent risk in patients with transient ischemic attacks. Radiomics based on medical imaging has been extensively used to predict prognosis, pathological type, response to treatment, and so on (35–37). Our previous study investigated the clinical radiomics nomogram for predicting ischemic stroke prognosis with good performance (3). Radiomics extracts high throughput quantitative features by combining radiology and machine learning, which can reflect the heterogeneity of lesions. Few studies explored radiomics based on DWI to predict stroke recurrence. Our study demonstrated that the DWI-based radiomics model could predict stroke recurrence with good performance. Stroke may present with or without underlying arterial pathologies because of the heterogeneity of stroke (14). Recent research demonstrated that lesions on DWI were indicators of microvascular occlusive events that were predisposing to microvessel rupture (19).

This study randomly selected 100 volumes by manual annotation and then used 2D U-net architecture to implement infarction lesions automatic segmentation, which was a recommended and efficient segmentation technique (24). 2D U-net convolutional neural networks for automated segmentation, an “end to end” segmentation method with robust accuracy performance, apply what is known as long-skip connections to directly connect opposing layers from contracting to the expanding paths (23, 38). A total of 513 radiomics features were extracted, including texture, intensity-related features, and wavelet features. However, a large amount of redundancy existed in the extracted features. A sparse representation feature selection model was used in our study to reduce redundancy and the risk of overfitting in subsequent classification, which was reported to apply in a previous study (27). In our study, the classification process involved RNN. RNN based on recursive connection is powerful in modeling temporal dynamics and learning appropriate feature representations (28). RNN model has demonstrated application with good performance in various medical tasks including genomic analysis, medical diagnosis, and recognizing patterns in sequential data (39). RNN can further improve the classification performance of the network by making the neurons in the hidden layer communicate with each other by storing the output results (24, 40).

## Limitations

There were several limitations in this research. First, due to a loss of follow-up cases in our retrospective study, potential selection bias was unavoidable. Second, we did not keep track of medication details after discharge; instead, we only kept track of whether the patients had taken their medications. Finally, our radiomic-based prediction model performed well in predicting stroke recurrence. However, the corresponding clinical implications of each feature were unclear, and further exploration is needed. Nevertheless, these encouraging results provide a noninvasive method for predicting ischemic stroke recurrence.

## Conclusion

In conclusion, we found that the radiomics-based DWI prediction model performed well in terms of predicting stroke recurrence. Our robust prediction performance was aided by machine learning and deep learning algorithms such as U-net architecture automatic segmentation, sparse representation feature selection, and RNN classification.

## Data availability statement

The raw data supporting the conclusions of this article will be made available by the authors, without undue reservation.

## Ethics statement

The local institutional committee on human research approved the retrospective study and waived the requirement for written informed consent due to its retrospective nature.

## Author contributions

YZ and BS designed this study. YS and JZ conducted the study and collected important data. HW drafted the manuscript. All authors contributed to the article and approved the submitted version.

## Funding

This research was funded by Natural Science Foundation of Minhang Hospital, Fudan University (2022MHBJ04 and 2022MHPY04).

## Conflict of interest

The authors declare that the research was conducted in the absence of any commercial or financial relationships that could be construed as a potential conflict of interest.

## Publisher's note

All claims expressed in this article are solely those of the authors and do not necessarily represent those of their affiliated organizations, or those of the publisher, the editors and the reviewers. Any product that may be evaluated in this article, or claim that may be made by its manufacturer, is not guaranteed or endorsed by the publisher.

## Supplementary material

supplementary table e-1 Magnetic resonance sequences parameters.

Supplementary Table e-2 The category and name of extracted radiomics features.

Supplementary Table e-3 The category and name of all selected radiomics features.

## Abbreviations

ADC: apparent diffusion coefficient
AIS: acute ischemic stroke
AUC: area under the curve
DWI: diffusion-weighted imaging
LAA: large artery atherosclerosis patients
MRI: magnetic resonance imaging
mRS: modified Rankin Scale
NIHSS: National Institutes of Health Stroke Scale
RNN: recurrent neural network
SAO: small artery occlusion
TOAST: Trial of Org 10172 in Acute Stroke Treatment.
