# Supplementary material for: Diffusion-weighted imaging-based radiomics for predicting 1-year ischemic stroke recurrence
Source: Front Neurol. 2022 Oct 28;13:1012896. doi: 10.3389/fneur.2022.1012896 (PMC9649925; doi:10.3389/fneur.2022.1012896)
Supplement: supplementary table e-1 — Magnetic resonance sequences parameters. [file Data_Sheet_1.docx]

**Table e-1 Magnetic Resonance Sequences Parameters**

| **Scanner** | **Sequence** | **TR (ms)** | **TE (ms)** | **Thickness (mm)** | **Spacing (mm)** | **FOV (mm*mm)** | **Matrix** |
| --- | --- | --- | --- | --- | --- | --- | --- |
| **MR 1** | Axial T1WI | 2259 | 25.4 | 5 | 1.5 | 240 *240 | 256*192 |
|  | Axial T2WI | 5582 | 111 | 5 | 1.5 | 240 *240 | 256*192 |
|  | Axial Flair | 8589 | 88.8 | 5 | 1.5 | 240 *240 | 256*192 |
|  | Axial DWI | 3203 | 83.9 | 5 | 1.5 | 240 *240 | 96*96 |
| **MR 2** | Axial T1WI | 2048 | 11.96 | 5 | 1.5 | 230 *230 | 288*192 |
|  | Axial T2WI | 4107 | 88.2 | 5 | 1.5 | 230 *230 | 288*192 |
|  | Axial Flair | 7500 | 96.66 | 5 | 1.5 | 230 *230 | 288*192 |
|  | Axial DWI | 2800 | 75.4 | 5 | 1.5 | 230 *230 | 128*128 |

TR: repetition time, TE: echo time, FOV: field of view

**Table e-2 The category and name of extracted radiomics features**

| **Category** | **Features name** | | | | | | | **Features number** | |
| --- | --- | --- | --- | --- | --- | --- | --- | --- | --- |
| **Intensity** |  | | | | | | | **18** | |
|  | energy | | | h-energy | kurtosis | max | |  | |
|  | mean absolute deviation | | | mean | media | min | |  |  |
|  | range | | | root mean square | skewness | standard-deviation | |  |  |
|  | h-uniformity | | | variance | h-mean | h-variance | |  |  |
|  | h-skewness | | | h-kurtosis |  |  | |  |  |
| **Texture** |  | | | | | | | **39** | |
| **GLCM** | energy | | | contrast | correlation | homogeneity | |  | |
|  | variance | | | sun average | entropy | dissimilarity | |  |  |
|  | short run emphasis | | | | long run emphasis | | |  |  |
| **GLRLM** | gray-level nonuniformity | | | | run-length nonuniformity | | |  |  |
|  | run percentage | | | | low gray-level run emphasis | | |  |  |
|  | high gray-level run emphasis | | | | short run low gray-level emphasis | | |  |  |
|  | short run high gray-level emphasis | | | | long run low gray-level emphasis | | |  |  |
|  | long run high gray-level emphasis | | | | gray-level variance | | |  |  |
|  | run-length variance | | | | small zone emphasis | | |  |  |
|  | large zone emphasis | | | |  | | |  |  |
| **GLSZM** | gray-level nonuniformity | | | | zone-size nonuniformity | | |  |  |
|  | zone percentage | | | | low gray-level zone emphasis | | |  |  |
|  | high gray-level zone emphasis | | | | small zone low gray-level emphasis | | |  |  |
|  | small zone high gray-level emphasis | | | | large zone low gray-level emphasis | | |  |  |
|  | large zone high gray-level emphasis | | | | gray-level variance | | |  |  |
|  | zone-size variance | | | |  | | |  |  |
| **NGTDM** | coarseness | contrast | busyness | | complexity | | strength |  |  |

GLCM: gray-level co-occurrence matrix，GLRLM: gray-level run-length matrix, GLSZM: gray-level size zone matrix, NGTDM: neighborhood gray-tone difference matrix
